# Phylogenetic Patterns of Swainsonine Presence in Morning Glories

**DOI:** 10.3389/fmicb.2022.871148

**Published:** 2022-05-03

**Authors:** Quynh N. Quach, Dale R. Gardner, Keith Clay, Daniel Cook

**Affiliations:** ^1^Department of Ecology and Evolutionary Biology, Tulane University, New Orleans, LA, United States; ^2^United States Department of Agriculture – Agricultural Research Service, Poisonous Plant Research Laboratory, Logan, UT, United States

**Keywords:** swainsonine, fungal symbiosis, morning glory, heritable symbiosis, *Ipomoea*

## Abstract

Endosymbionts play important roles in the life cycles of many macro-organisms. The indolizidine alkaloid swainsonine is produced by heritable fungi that occurs in diverse plant families, such as locoweeds (Fabaceae) and morning glories (Convolvulaceae) plus two species of Malvaceae. Swainsonine is known for its toxic effects on livestock following the ingestion of locoweeds and the potential for pharmaceutical applications. We sampled and tested herbarium seed samples (*n* = 983) from 244 morning glory species for the presence of swainsonine and built a phylogeny based on available internal transcribed spacer (ITS) sequences of the sampled species. We show that swainsonine occurs only in a single morning glory clade and host species are established on multiple continents. Our results further indicate that this symbiosis developed ∼5 mya and that swainsonine-positive species have larger seeds than their uninfected conspecifics.

## Introduction

Heritable micro-organisms, passed down from mother to offspring through seeds or eggs, play critical roles in the life cycles of many organisms but their prevalence and functional roles are unknown for most plants. This stands in contrast with the well-documented ancient endosymbiotic origins of chloroplasts and mitochondria. Differing from the diverse endophytic fungal communities in plants that result from the environmental transmission and form highly localized infections within single leaves or other tissues (Christian et al., 2015), heritable micro-organisms, most often fungal endosymbionts, are associated with only a few selected major plant families. When a vertically transmitted symbiont benefits the host, it has an indirect positive effect on its own fitness as hereditary symbionts are completely dependent on the host for their own propagation and reproductive fitness and so should not reduce host fitness (Ewald, 1987; Lipsitch et al., 1996). Understanding the origin, distribution, and function of heritable fungi provide insights into the evolution and ecology of major plant groups.

**FIGURE 1:**
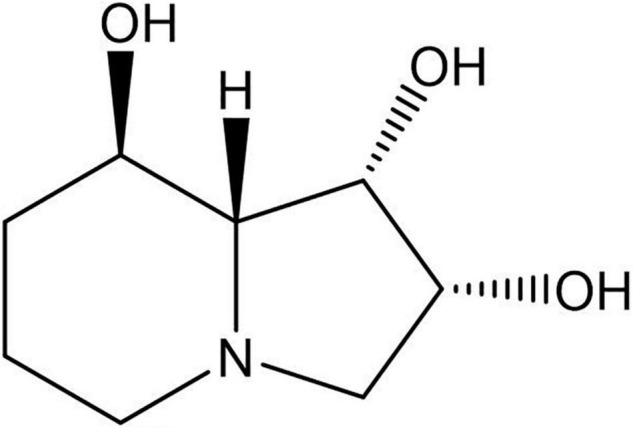
Structure of the indolizidine alkaloid swainsonine.

Fungi in general produce a wide array of secondary metabolites. The production of bioactive metabolites by endosymbiotic fungi that is observed in multiple hereditary plant-endosymbiotic fungal associations might have particular importance to the fitness of both the fungus and the host species. The benefits and costs of the symbiosis are not well understood in these systems except for the fact that many of these metabolites have toxic effects upon ingestion of the host plant. The indolizidine alkaloid swainsonine (Figure 1) is the toxic principle in a number of plant species worldwide and causes severe toxicosis in livestock grazing these plants (Colegate et al., 1979; Molyneux and James, 1982; Molyneux et al., 1995; de Balogh et al., 1999; Colodel et al., 2002; Dantas et al., 2007). Swainsonine is an alpha-mannosidase and mannosidase II inhibitor that alters glycoprotein processing and causes lysosomal storage disease (Colegate et al., 1979; Dorling et al., 1980; Tulsiani et al., 1988). Consumption of swainsonine-containing plants by grazing animals leads to a chronic disease characterized by weight loss, depression, altered behavior, decreased libido, infertility, and death, which are estimated to cause tens of millions of dollars in livestock losses annually (Panter et al., 1999). Swainsonine occurs sporadically in three diverse plant families: Fabaceae (Fabales), Malvaceae (Malvales), and the morning glory family Convolvulaceae (Solanales) (Cook et al., 2014).

Many of the plant species identified to date that contain swainsonine primarily result from episodes of poisoning as the clinical signs and associated pathology are similar in poisoned livestock (Cook et al., 2014). These cases of livestock poisoning include more than 25 swainsonine-containing legumes commonly referred to as locoweeds (Fabaceae), such as *Astragalus*, *Oxytropis*, and *Swainsona* species. Toxic species within these genera have been identified in Australia, China, North America, and South America. Likewise, seven morning glory species belonging to the genus *Ipomoea* have been reported to contain swainsonine due to episodes of livestock poisoning in Africa, Australia, and South America (Cook et al., 2014). Lastly, two swainsonine-containing *Sida* species (Malvaceae) have been identified in South America due to poisoning of livestock (Colodel et al., 2002; Micheloud et al., 2017). More recently, preserved herbarium specimens have been used to test and identify several *Astragalus*, *Oxytropis*, and *Swainsona* species that contain swainsonine (Cook et al., 2016, 2017b,c,d). Herbarium specimens provide an excellent resource to investigate the phytochemical composition of plants and their distribution among species (Cook et al., 2021).

Prior research has demonstrated that all swainsonine-containing plant taxa investigated to date are associated with systemic, heritable, seed-transmitted fungal symbionts that produce swainsonine. Swainsonine-containing legumes (*Astragalus, Oxytropi*s, and *Swainsona* spp.) are associated with a fungal symbiont from *Alternaria* section *Undifilum* (Pleosporales) (Braun et al., 2003; Yu et al., 2010; Baucom et al., 2012; Grum et al., 2013). By contrast, the swainsonine-containing convolvulaceous taxon, *Ipomoea carnea* is associated with a distinct fungal symbiont belonging to the order Chaetothyriales (Cook et al., 2013 and Supplementary Figure 1). Other phylogenetically disjunct groups of swainsonine-producing fungi are found to associate with distantly related plant families. *Metarhizium* sp. (Hypocreales) are both insect pathogens and plant symbionts while *Slafractonia leguminicola* (Pleosporales), a pathogen of red clover (*Trifolium pratense*), also produce swainsonine (Gough and Elliott, 1956; Cook et al., 2014). Swainsonine-producing fungi that are not associated with plants include several species in the Arthrodermataceae (Onygenales), a family of fungi that causes athlete’s foot and ringworm diseases in humans and other mammals (Cook et al., 2017a). Recently, molecular genetic studies have identified and characterized the orthologous swainsonine biosynthetic genes in the above four orders of fungi (Cook et al., 2017a).

It is notable that other species of morning glories (Convolvulaceae) are associated with a distinct group of hereditary fungi classified in the genus *Periglandula* (Clavicipitaceae), that produce the ergot and indole diterpene alkaloids similar to those found in grasses infected with fungal endophytes from the same fungal family (Steiner et al., 2011; Schardl et al., 2013). A recent study found that *Periglandula* fungi are associated with particular clades of morning glories, where they produce distinct mixtures of ergot alkaloids and where symbiotic hosts have significantly larger seed sizes than non-symbiotic species (Beaulieu et al., 2021).

To further our understanding of the morning glory/Chaetothyriaceae/swainsonine association and to test the hypothesis that the presence of swainsonine, used here to indicate the presence of the heritable symbiont, is found in particular phylogenetic groups, we (1) tested for the presence of swainsonine in seeds of 244 morning glory species from diverse, worldwide herbarium collections in order to determine the global distribution and diversity of Chaetothyriaceae symbiosis, (2) obtained phylogenetically informative internal transcribed spacer (ITS) sequences from GenBank and determined the distribution of swainsonine-positive (S+) and S − species across the morning glory host phylogeny to elucidate the evolutionary history of the symbiosis, and (3) evaluated whether swainsonine and Chaetothyriaceae symbiosis vary with a key plant life-history trait to understand potential selective benefits of this symbiosis for the host. Our results provide new insights into the evolution of heritable fungal symbiosis and the diversification of swainsonine-producing fungi in morning glories.

## Materials and Methods

### Herbarium Survey

**FIGURE 2:**
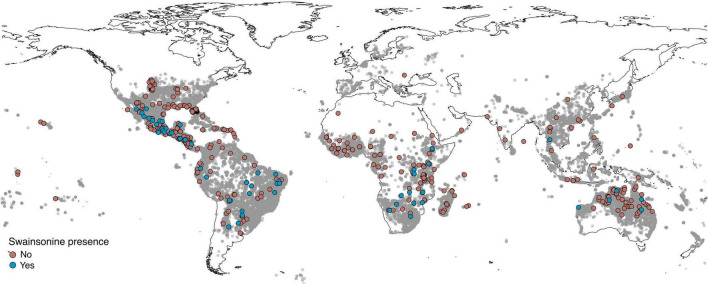
Distribution of samples. Global distribution of samples with geographic information (*n* = 666) tested for swainsonine. Gray circles represent herbarium specimens from *Argyreia, Ipomoea, Stictocardia*, and *Turbina* (now *Ipomoea*) species from the Global Biodiversity Information Facility (GBIF.org, 2021a,b,c,d) to illustrate the geographical distribution of the tribe Ipomoeeae with higher concentrations of sample in darker areas.

We obtained permissions to sample mature seeds and/or vegetative parts from herbarium specimens from the Missouri Botanical Garden (MOBOT), Vadense Herbarium (WAG, Netherlands), Western Australian Herbarium (PERTH), and the Australian National University Herbarium (ANU), in addition to seeds from personal collections of Convolvulaceae researchers. We sampled mature seeds and/or vegetative parts from a total of 244 morning glory species from 983 herbarium sheets, where species identity, mean swainsonine content, mean seed mass, and mean collection latitude are reported by species for each herbarium specimen where available (Supplementary Data 1). The sampled species include representatives from all continents except Europe (where we had only one sample; Figure 2) and Antarctica. Some species have localized distributions, while others have regional or pantropical distributions (Supplementary Data 1). Our samples represented a subset of all potential host species because most herbarium specimens examined did not have mature seeds. We relied on the records of herbarium specimen labels but realize that misidentifications are possible and have updated any naming conventions to the best of our abilities.

### Swainsonine Analysis

Swainsonine was extracted using a modification of a procedure described by Gardner and Cook (2011). A measured quantity of plant material was extracted in a volume of 2% acetic acid for 18 h with agitation. After extraction, samples were centrifuged and an aliquot from the extraction was diluted into 20 mM ammonium acetate in a 1 ml auto-sampler vial. Samples were analyzed by liquid chromatography with tandem mass spectrometry (LC-MS/MS) to detect swainsonine as previously described (Gardner et al., 2001). Presence/absence data were recorded primarily with concentrations being recorded when the sample permitted.

Species that tested positive for swainsonine by the LC-MS/MS method were subsequently verified to contain swainsonine by gas chromatography-mass spectrometry (GC-MS) as a secondary screen. Sample preparation was similar to those described by Cook et al. (2016). All samples were analyzed by GC-MS for swainsonine (trimethylsilyl [TMS] derivative) using the GC-MS conditions previously described (Gardner et al., 2001).

### Estimating Morning Glory Phylogeny With ITS Sequence Data

We downloaded the ITS sequence entries from GenBank available in 2021 for 201 species (Supplementary Data 2) across multiple genera (*Argyreia, Distimake, Ipomoea, Jacquemontia, Stictocardia*, and *Merremia*) that we sampled for swainsonine. For each species, we obtained the complete ITS1 + 5.8S rRNA + ITS2 sequence where possible. In some cases, this was obtained by combining partial sequences from multiple accessions of the same species. In total, we obtained 194 complete ITS1 + 5.8S + ITS2 sequences, three sequences from combining partial sequences and four were partial ITS1 +5.8S + ITS2 sequences. These sequences were aligned and trimmed using MAFFT v7.489 (Katoh et al., 2019) using the L-INS-i alignment strategy with a 1.7 gap penalty. We used jModelTest2 (Darriba et al., 2012) on CIPRES Science Gateway (Miller et al., 2010) to determine the best substitution model, which was GTR+G+I based on Akaike Information Criteria (AIC). A maximum-likelihood phylogeny was then estimated using RAxML-HPC2 on XSEDE on CIPRES Science Gateway (Miller et al., 2010) using the GTR+G model, as the developers of RAxML do not recommend using the proportion of invariable sites estimation, with 1,000 bootstrap replicates. We also estimated a Bayesian tree using MrBayes v3.2.7a on XSEDE on CIPRES Science Gateway (Miller et al., 2010) with two runs and four independent chains using the GTR+G+I model for 10,000,000 generations, sampled every 1,000 generations, with the first 25% as burn in. We used Tracer v1.7.2 (Rambaut et al., 2018) to check for convergence. For both methods, we designated *Merremia sibirica* as the outgroup species based on previous Convolvulaceae phylogenetic work (Simoes et al., 2015).

We recognize the limitations of inferring a phylogeny based on a single gene, but most of our sampled species had no other gene sequences available. Our results are in general agreement with published smaller, multi-gene phylogenies, and the ITS sequences used here have been used to construct phylogenies in other published studies (Muñoz-Rodríguez et al., 2019; Beaulieu et al., 2021).

### Testing Phylogenetic Signal for Swainsonine and Seed Mass

i) Swainsonine presence: We tested for phylogenetic signal in the presence of swainsonine to determine whether S+ species across the Ipomoeeae tribe are closely related species. We followed the analyses methods described by Beaulieu et al. (2021). In short, we used the function “fitDiscrete” from the R package “geiger” (Pennell et al., 2014) to fit different models of character evolution (FitzJohn et al., 2009). We used the all-rates-different (ARD) model based on the lowest AIC score. We then used Pagel’s λ (Pagel, 1999) to assess the phylogenetic signal where there is a strong phylogenetic signal if λ is equal to or near 1. We transformed our phylogeny to one with no phylogenetic signal (λ = 0) and compared its likelihood scores with our observed phylogeny and computed the *p*-value to determine if our observed phylogeny significantly differed from the no-signal phylogeny.

We also computed Fritz and Purvis’s D (FPD) (Fritz and Purvis, 2010) as an alternative measure of the phylogenetic signal using the function “phylo.d” from R package “caper” (Orme et al., 2013). Where D is a measure of phylogenetic dispersion and can be less than 1 (non-random distribution) or equal to 1 (random distribution).

ii) Seed mass: All average seed masses were log10-transformed to normalize the data then phylogenetic signal testing for seed mass was conducted as was done for the presence of swainsonine, except the “fitContinuous” function from the R package “geiger” (Pennell et al., 2014) was used since the data are continuous. The FPD statistic can only be applied to binary traits so we did not apply it to seed mass.

### Ancestral Character-State Reconstruction

We followed the analyses methods described by Beaulieu et al. (2021). In short, we reconstructed ancestral states for the presence of swainsonine (yes, no) and seed mass (large, small) using the R packages “ape” (Paradis and Schliep, 2019) and “phytools” (Revell, 2012). We used “ace” from R package “ape” to fit the best model of trait evolution for our data by comparing AIC scores, which show that the ARD model was better than other models (equal-rates and symmetric). This produces a likelihood at each internal node of being S+ or S −, or large or small seeds, character states. Ancestors were designated as being one state or another if the likelihood of it being that state was greater than 75%.

### Correlated Evolution in Swainsonine Presence, Concentration, and Seed Mass

We followed the analyses methods described by Beaulieu et al. (2021). In short, we used two methods to test for correlated evolution between swainsonine and seed mass. First, using log10-transformed seed mass data, a phylogenetic logistic regression was performed using the function “phyloglm” from the R package “phylolm” (Ho and Ané, 2014). Second, the function “fitPagel” from the R package “phytools” (Revell, 2012) was used to perform Pagel’s test of correlation (Pagel, 1994). Pagel’s correlation assesses correlated evolution between two binary characters, so we transformed seed mass into a binary trait. The average seed mass of all surveyed species was log10-transformed and was used to separate the large (> 1.615, 41.23 mg) from the small (< 1.615, 41.23 mg) group.

In addition to a linear regression, we use the function “phylolm” from the R package “phylolm” (Ho and Ané, 2014) to perform a phylogenetic linear regression to test for a correlation between seed mass and swainsonine concentration in S+ species (*n* = 21) where both values were log10-transformed.

## Results

### Distribution of Swainsonine in Morning Glory Species

In total, 32 of 244 (13%) morning glory species that we evaluated from herbarium specimens (Supplementary Data 1) contained swainsonine and are therefore symbiotic (Figure 2). In total, 24 of the 32 species were not previously known to be S+, and S+ species were distributed across multiple continents (Table 1). *Ipomoea sericosepala* was previously reported to be S+ (Mendonça et al., 2018) but we did not detect swainsonine in our samples of the species. Given that there are more than 800 morning glory species, the total number of S+ species will certainly become larger with further sampling.

**TABLE 1:** Swainsonine positive species.

Swainsonine positive	Location	References	Average seed mass (mg)	Swainsonine concentration (%)
*Ipomoea albivenia*	Africa		71.5	1.07
*Ipomoea arborescens*	Mexico		52.5	0.23
***Ipomoea brasiliana^1^***	S. America	Dantas et al., 2007; Mendonça et al., 2012, 2018	108	0.5
***Ipomoea calobra^1^***	Australia	Molyneux et al., 1995	N/A	0.11
***Ipomoea carnea^1^***	Pantropical	de Balogh et al., 1999; Haraguchi et al., 2003	66.15	0.07
*Ipomoea cavalcantei*	S. America		20	0.06
*Ipomoea chilopsidis*	Mexico		N/A	N/A
***Ipomoea costata^1^***	Australia	Cook et al., 2019	114	0.09
*Ipomoea cuneifolia*	S. America		10	0.12
*Ipomoea descolei*	S. America		58	0.4
*Ipomoea haenkeana*	S. America		8	0.07
***Ipomoea hieronymi^1^***	S. America	Cholich et al., 2021	33	0.25
*Ipomoea intrapilosa*	Mexico		79	0.04
*Ipomoea lapidosa*	Africa		123	0.79
*Ipomoea longifolia*	C. America		117.5	0.1
*Ipomoea malvaeoides*	S. America		30	0.08
*Ipomoea marcellia*	S. America		N/A	N/A
*Ipomoea marmorata*	Africa		213	0.45
***Ipomoea megapotamica^1^***	S. America	Barbosa et al., 2006	N/A	N/A
*Ipomoea murucoides*	C. America		154.29	0.14
*Ipomoea opulifolia*	S. America		56	N/A
*Ipomoea pauciflora*	C. & S. America		73.6	0.14
***Ipomoea polpha^1^***	Australia	Molyneux et al., 1995	99	N/A
*Ipomoea populina*	C. America		65	N/A
*Ipomoea prismatosyphon*	Africa		71	N/A
***Ipomoea pterocaulis^1^***	S. America	Barbosa et al., 2006	20	0.74
***Ipomoea rosea^3^***	S. America	Mendonça et al., 2018	N/A	N/A
*Ipomoea saopaulista*	S. America		4.25	0.03
***Ipomoea sericosepala^2^***	S. America	Mendonça et al., 2018	44.33	N/A
*Ipomoea sumatrana*	Asia		N/A	N/A
*Ipomoea valenzuelensis*	S. America		N/A	N/A
*Ipomoea verbascoidea*	Africa		56.17	0.67
*Ipomoea wolcottiana*	C. & S. America		34.5	0.32
*Ipomoea yardiensis*	Australia		N/A	N/A
***Jacquemontia corymbulosa^3^***	S. America	Mendonça et al., 2018	N/A	N/A

*Bolded species were previously reported to contain swainsonine; non-bolded reported here for the first time. All species were analyzed in this study unless noted otherwise. N = North, C = Central, S = South.*

*^1^Species confirmed to contain swainsonine in this survey.*

*^2^Species was confirmed to be negative through this survey.*

*^3^This species was not investigated in this survey.*

### Swainsonine Distribution in Host Phylogeny

**FIGURE 3:**
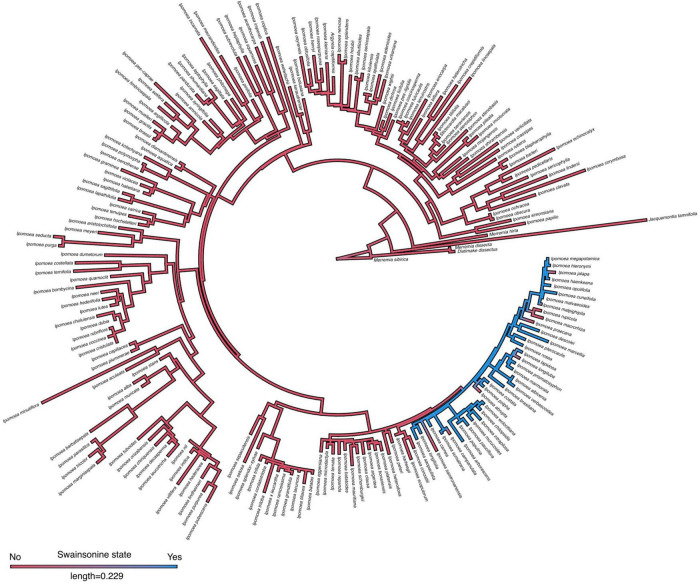
Internal transcribed spacer (ITS) phylogeny of morning glories. Maximum likelihood ITS phylogeny of morning glories (*n* = 201) with ancestral state reconstruction of swainsonine presence by density map based on 1,000 stochastic character maps. Branch color represents the probability of the character state. The length of the legend equals units of substitution per site.

**FIGURE 4:**
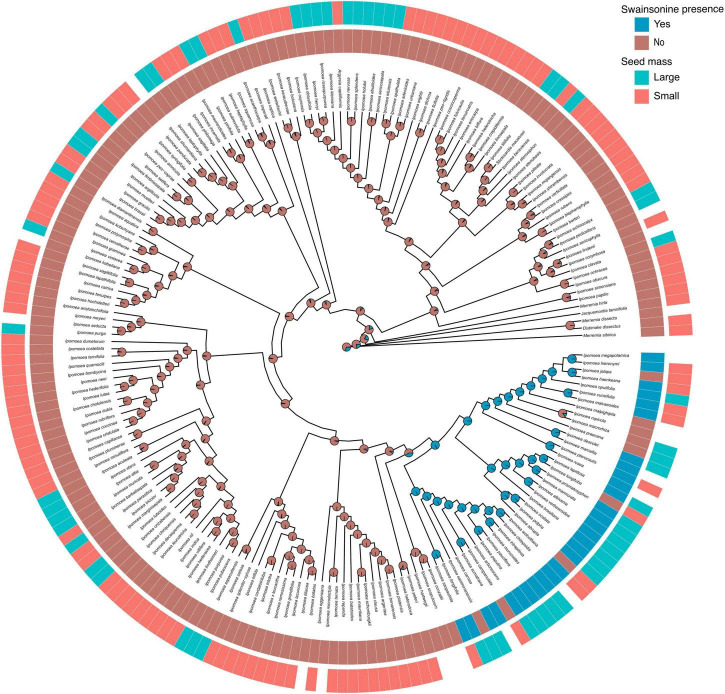
Ancestral state reconstruction of swainsonine presence. Phylogenetic distribution of swainsonine presence and seed mass. Species lacking seed mass data are represented by blank spaces. Pie charts at nodes indicate the relative likelihoods of the swainsonine character state.

To evaluate the occurrence of swainsonine in relation to morning glory host phylogeny, we obtained published ITS sequences for 201 of our 244 surveyed species (Supplementary Data 2). In both our maximum likelihood and Bayesian inferred phylogenies (Figures 3, 4 and Supplementary Figure 2), we found that all S+ species occurred within a single clade of closely related species and that swainsonine was absent from the rest of the morning glory phylogeny. We found a significant phylogenetic signal for the presence of swainsonine (Pagel’s λ = 0.9, *D* = − 0.67, *p* < 0.001), suggesting that species with swainsonine are more closely related than expected by chance (Figures 3, 4). Our results suggest that the oldest common ancestor observed was S − and that this symbiosis has arisen relatively recently within the morning glory phylogeny given that the S+ clade was the last to split from the lineage (Figures 3, 4). This pattern also suggests that the symbiosis is strictly heritable and vertically transmitted from a single S+ ancestor, especially given the wide geographic distribution of S+ species. However, several S − species occur within this primarily S+ clade, suggesting that the symbiosis can be lost.

### Seed Mass and Swainsonine

**FIGURE 5:**
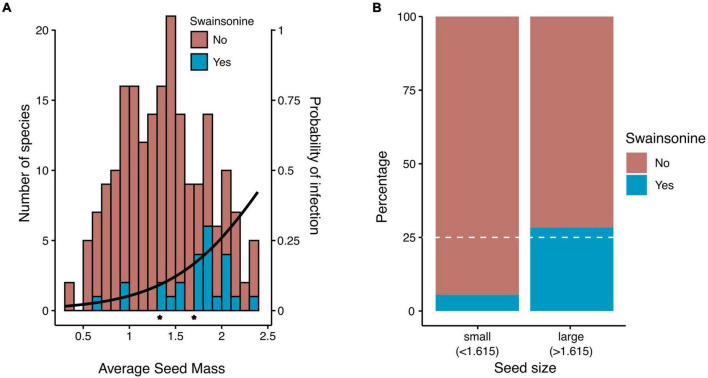
Relationship of seed mass and swainsonine. Seed mass is log10-transformed. **(A)** The number of species with (*n* = 34) or without (*n* = 210) swainsonine by mean seed mass. Stars along the x-axis indicate the mean seed mass of each group. The binomial regression line shows the probability of infection based on mean seed mass. **(B)** Proportion of small and large-seeded species with or without swainsonine. Dashed lines represent the expected number of swainsonine species if the proportion of S+ species is equivalent across seed mass.

Species with larger seeds are often larger as adults, longer-lived, and have lower growth rates than species with smaller seeds (Silvertown, 1981; Moles et al., 2005). Therefore, we measured seed mass as a proxy for plant life history strategy. We found a significant phylogenetic signal in seed mass (λ = 0.89, *p <* 0.001), suggesting that closely related species have more similar seed mass. Furthermore, seeds of S+ species were approximately two times larger than seeds of S − species (69.5 vs. 37.3 mg, t(32.6) =, *p* < 0.005, Figure 5A). When we transformed seed mass to a binary state, we found a significantly higher proportion of species with large seeds containing swainsonine than species with small seeds (*X*^2^ = 18.4, *p* < 0.001, Figure 5B, Supplementary Figure 3). We then performed Pagel’s test for correlated evolution between two discrete characters after accounting for host phylogeny (Pagel, 1994) and found a significant positive correlation between large seed mass and swainsonine (*p* = 0.047, Figure 4). However, our phylogenetic logistic regression analysis (Ives and Garland, 2010), which tests for a correlation between discrete and continuous characters based on a phylogeny, showed that seed mass as a continuous variable is not a direct predictor of swainsonine presence. Finally, phylogenetic regression (Ho and Ané, 2014) did not show a significant correlation between seed mass and swainsonine concentration in S+ species (Supplementary Figure 4).

## Discussion

The results reported here show that all S+ *Ipomoea* species occur in just a single clade while the rest of the phylogeny is S −. Many of the S+ *Ipomoea* species reported here are part of Clade A1 described in Wood et al.’s (2020) monograph of *Ipomoea* species from the Americas. In fact, 22 of 26 species in clade A1 from the Americas surveyed here contained swainsonine. Species in this clade are perennial, generally have tuberous roots, and have distinctive pollen and floral traits (Wood et al., 2020). Within Clade A1, there are additional groups, for example, the Arborescens clade, which is composed of small trees, large shrubs, or lianas, where many are S+ that includes *I. pauciflora*, *I. arborescens*, *I. chilopsidis*, *I. intrapilosa*, *I. populina*, *I. wolcottiana*, and *I. murucoides*. These common morphological features within this clade might have played a role in the evolution of the swainsonine symbiosis.

This pattern of closely related S+ species observed here is similar to observations in the legume genus *Astragalus*, which like *Ipomoea* is species rich and morphologically and phylogenetically complex (Barneby, 1964; Wojciechowski et al., 1999). Unlike the phylogeny of *Ipomoea* reported by Muñoz-Rodríguez et al. (2019) and herein, the phylogeny of *Astragalus* generally has poor resolution with only a few well-defined clades due to the recency of the radiation (Wojciechowski et al., 1999). One exception is a phylogenetic clade composed of six North American and eight South American *Astragalus* species (Wojciechowski et al., 1999; Scherson et al., 2008). Surveys for S+ species of *Astragalus* identified swainsonine in 13 of these 14 species in this clade that share a common phylogenetic origin (Cook et al., 2017b,c). Among the more than 50 species of *Astragalus* that were reported to contain swainsonine in North and South America that includes the 13 mentioned above (Cook et al., 2017b,c), many share common morphological features, such as an inflated pod, as they co-occur in morphologically related taxonomic sections (Barneby, 1964).

Documentation of swainsonine-containing morning glories is relatively recent as compared to other swainsonine-containing legumes (Colegate et al., 1979; de Balogh et al., 1999). Our inferred phylogeny also suggests that the swainsonine symbiosis is a relatively recent plant-fungal association in the Convolvulaceae. We estimate the divergence age of the S+ clade to be approximately 5 mya based on the conclusions of a recently published time-calibrated nuclear phylogeny study of the family (Muñoz-Rodríguez et al., 2019). Further, all S+ species are closely related, which strongly suggest that the fungal endosymbiont is strictly transmitted vertically, consistent with observations on the transmission of the fungal endosymbiont of *I. carnea* (Cook et al., 2013). Furthermore, this pattern suggests that the symbiosis is transmitted vertically during the speciation process. Though S+ species that belong to the same morning glory clade, they are widely distributed geographically across Australia, Africa, Americas, and Southeast Asia, suggesting that these species originated from a common S+ ancestor. The occurrence of occasional S − species within the same clade suggests that the symbiosis has been occasionally lost in the lineage. These S − species also occur over multiple continents. It is highly likely that there are many other *Ipomoea* species that contain swainsonine given that our samples represent 26 of 127 species (20%) in Clade A1 described by Wood et al. (2020). Based upon our survey reported here, the frequency of S+ species in the clade is greater than 85% or over 100 species.

The Chaetothyriales symbiont associated with *I. carnea* has been shown to grow as an epibiont on the adaxial leaf surface associated with oil glands (Cook et al., 2013), similar to *Periglandula* on the adaxial surface of *Ipomoea asarifolia* (Steiner et al., 2011). Plants that lack swainsonine lack the corresponding epibiont on the leaf surface (Cook et al., 2013). The epibiont has only been detected growing on the leaf surface and has not been observed growing between cells in different plant tissues. Notably, there is no evidence of horizontal transmission as plants containing the epibiont and plants lacking the epibiont grown in proximity to each other in the greenhouse maintain their original chemical phenotype (Cook et al., 2013; Noor et al., 2021). It is not known whether the Chaetothyriales symbiont grows as an epibiont in other swainsonine-containing *Ipomoea* species, but should be examined. In *I. asarifolia*, *Periglandula* is associated with and is thought to feed on the oils secreted from the glandular trichomes. Potentially, an analogous type of association may be occurring with the Chaetothyriales symbiont wherein it benefits from the plant through its association with the glandular trichomes thus facilitating the plant host relationship. It is less clear what potential benefits the host plant obtains but theory suggests that there should be a benefit to the host plant.

Interestingly, S − species in the rest of the phylogeny include those that have been shown to contain ergot alkaloids produced by another heritable fungal endosymbiont (*Periglandula* sp.) found in morning glories, where 25% of species examined contained ergot alkaloids (Beaulieu et al., 2021). Thus, about 40% of all morning glory species examined thus far are symbiotic with one heritable fungus or the other. Swainsonine might affect plant growth in ways that were not captured in our study and this non-overlapping host species distribution of endosymbionts potentially reflects symbiont exclusion to reduce physiological costs to the host or redundancy in functions for the host. A previous study has shown that *Periglandula*-infected *I. tricolor* plants have reduced biomass when grown in isolation, but performed better when grown in the presence of root-knot nematodes as compared to non-infected conspecifics (Durden et al., 2019).

Similar to the *Periglandula* sp. symbiosis in the morning glory family (Beaulieu et al., 2021), S+ species have larger seeds than their S − congeners in our survey. Although the toxic properties of swainsonine can lead to poisoning of animals when ingested, a previous study did not find any evidence that animals avoid S+ plants (*Oxytropis* and *Ipomoea*) (Pfister et al., 2020). However, larger seeds represent a larger energy investment by the maternal plant, and swainsonine found in seeds might serve as a deterrent for other potential seed predators, such as birds or insects. For example, *Metarhizium* sp., which produces toxic metabolites, such as swainsonine, is widely used as an organic plant insecticide (Zimmermann, 2007; St Leger and Wang, 2010). Swainsonine may also increase the fitness of the seed by affecting other fungi associated with the seed or potential seed pathogens. Recent studies have shown that the presence of the heritable symbiont *Alternaria fulva* and associated swainsonine in the spotted locoweed, *Astragalus lentiginosus* (Fabaceae), alters the richness and diversity of other fungi associated with the plant (Harrison et al., 2018).

Greater seed mass has also been shown to be characteristic of long-lived plants (Silvertown, 1981) and many of the S+ species here are woody, perennial species (Wood et al., 2020). Larger seeds can increase seedling survival that would be required for the longer time that larger plants take to reach maturity (Moles and Westoby, 2004). Seed mass also correlates with the net primary productivity of the plant and some environmental conditions, such as precipitation (Moles et al., 2005), so it is possible that the correlation of swainsonine present with larger seed mass may reflect other factors than plant life history.

Swainsonine concentrations have been shown to vary between plant tissues, as a function of phenology, and due to endophyte genotype in the legume *Oxytropis sericea*. In *O. sericea*, little or no swainsonine was detected in the root or crown of the plant with greater concentrations found in above-ground parts (Cook et al., 2009). Concentrations have been also shown to vary in above-ground parts as a function of developmental age with the greatest concentrations as the plant reaches maturity (Cook et al., 2012). Swainsonine concentrations also vary by endophyte genotype within and between host species, which influences the concentrations found in plant tissues (Braun et al., 2003; Cook et al., 2013). The variation of swainsonine concentrations among different plant tissues and across developmental stages should be examined in different S+ *Ipomoea* species. Although we did not find a significant correlation between seed mass and swainsonine concentration in S+ species, there is a positive trend that might become stronger with increased sampling (*p* = 0.075, Supplementary Figure 4). Another interesting pattern is that African S+ species have higher concentrations than S+ species from other continents (Supplementary Figure 4). This group of African species forms a subclade within the S+ clade and their high swainsonine concentrations could suggest a difference in endosymbiont genotypes, consistent with observations in *Astragalus* and *Oxytropis* species (Braun et al., 2003; Cook et al., 2013).

Although our study comprised only a fraction (30%) of all described *Ipomoea* species, it demonstrates the distribution and heritability of swainsonine-producing fungi in morning glory species. The closely related group of S+ morning glories presents a promising future model system for studying heritable fungal symbiosis and its evolution. One important question is whether the diversity and density of other horizontally acquired fungal endophytes in the host microbiome vary with the presence of the heritable symbiont and its swainsonine chemistry as had been shown in other host species (Harrison et al., 2018). In addition, experimental performance and functional studies of S+ vs. S − morning glory species would provide further insights into the costs and benefits of this symbiosis. The genetic diversity and relatedness of the various orders of swainsonine-producing fungal endosymbionts in different plant families also pose interesting directions for studying the origins of these chemically related symbioses, whether these distinct symbioses have similar effects on host plants from different families and fungal-host specificity.

## Data Availability Statement

The original contributions presented in the study are publicly available. This data can be found here: doi: 10.6084/m9.figshare.19010885.

## Author Contributions

DC, QQ, and KC conceptualized the study. DC, DG, and KC collected and generated data. QQ performed statistical analyses with input from KC. All authors contributed to writing, reviewing, and editing the final manuscript.

## Conflict of Interest

The authors declare that the research was conducted in the absence of any commercial or financial relationships that could be construed as a potential conflict of interest.

## Publisher’s Note

All claims expressed in this article are solely those of the authors and do not necessarily represent those of their affiliated organizations, or those of the publisher, the editors and the reviewers. Any product that may be evaluated in this article, or claim that may be made by its manufacturer, is not guaranteed or endorsed by the publisher.

## Abbreviations

(S+): Swainsonine positive
(S−): swainsonine negative.
