# Supplementary material for: Phylogenetic Patterns of Swainsonine Presence in Morning Glories
Source: Front Microbiol. 2022 May 3;13:871148. doi: 10.3389/fmicb.2022.871148 (PMC9111539; doi:10.3389/fmicb.2022.871148)
Supplement: Supplementary file 1 [file Data_Sheet_1.docx]

**Supplementary Materials for “Phylogenetic patterns of swainsonine presence in morning glories”**

Quynh N. Quach, Dale R. Gardner, Keith Clay, and Daniel Cook^*^

* Correspondence: [daniel.cook@usda.gov](mailto:daniel.cook@usda.gov)

**1 Supplementary Data**

All Supplementary Data is available at figshare DOI: 10.6084/m9.figshare.19010885.

**Supplementary Data 1**. All herbarium specimens used in this study.

**Supplementary Data 2.** All accession numbers of ITS sequences used in the study.

**2 Supplementary Figures**


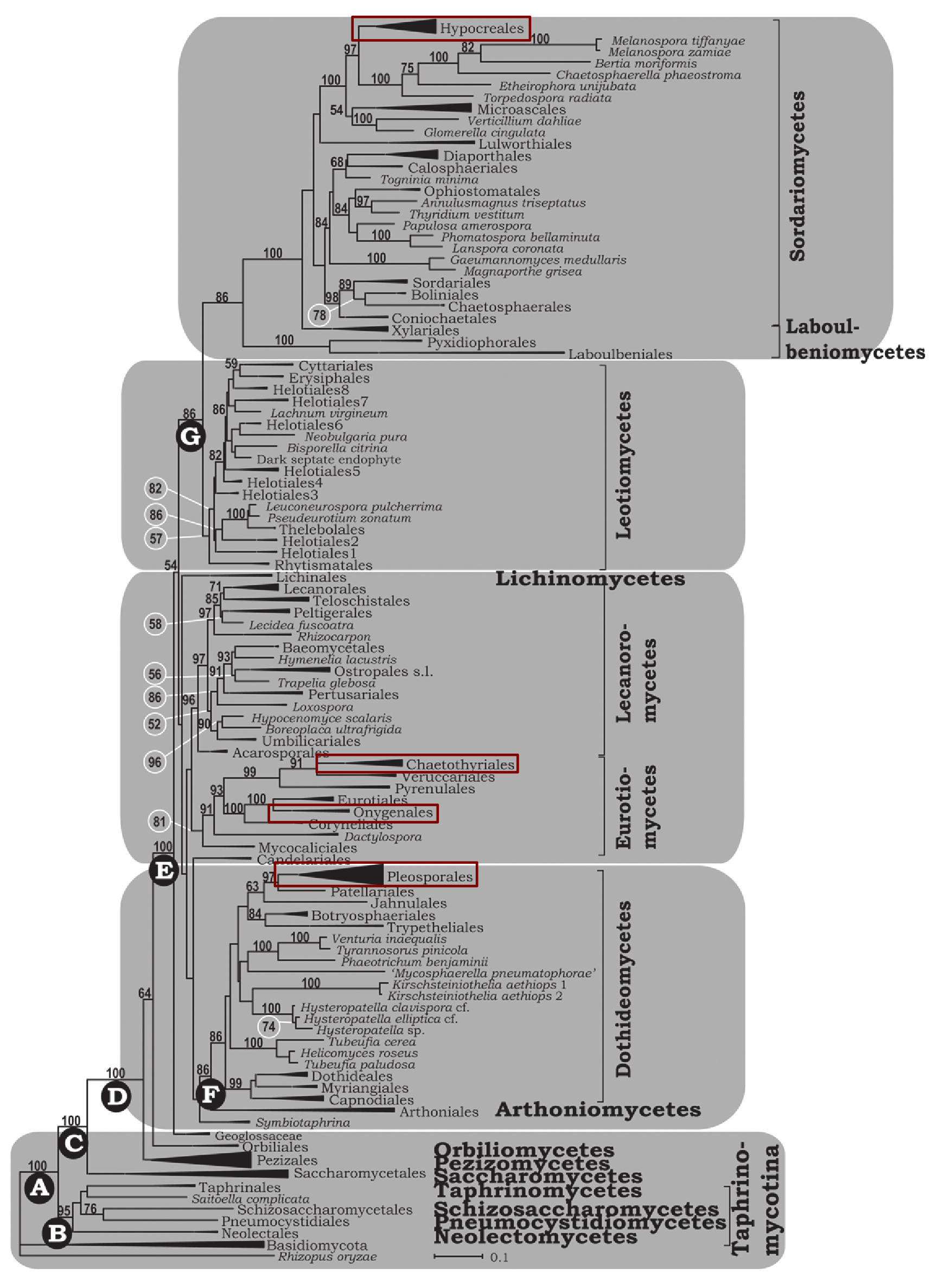


**Supplementary Figure 1.** Modified Ascomycota phylogeny from Figure 3 of Schoch et al. [(2009)](https://paperpile.com/c/qu804x/5JCRn). Red boxes denote the four orders of fungi which have been found to produce swainsonine in their plant hosts.


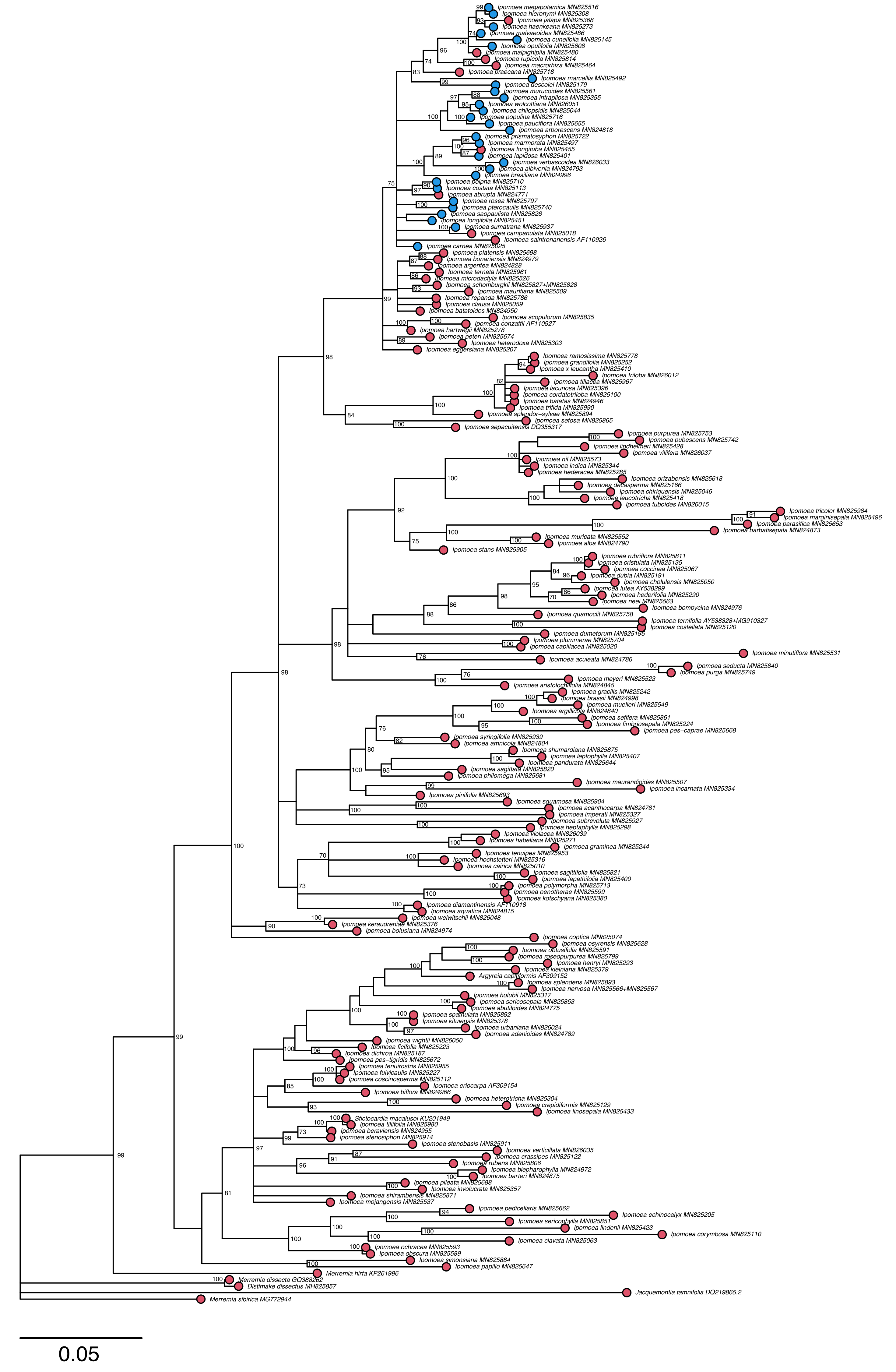


**Supplementary Figure 2.** Bayesian phylogeny of morning glories (n=201) based on ITS sequences showing the current state of swainsonine symbiosis. Node labels indicate posterior probabilities. Scale bar corresponds to expected number of substitutions per site.

^
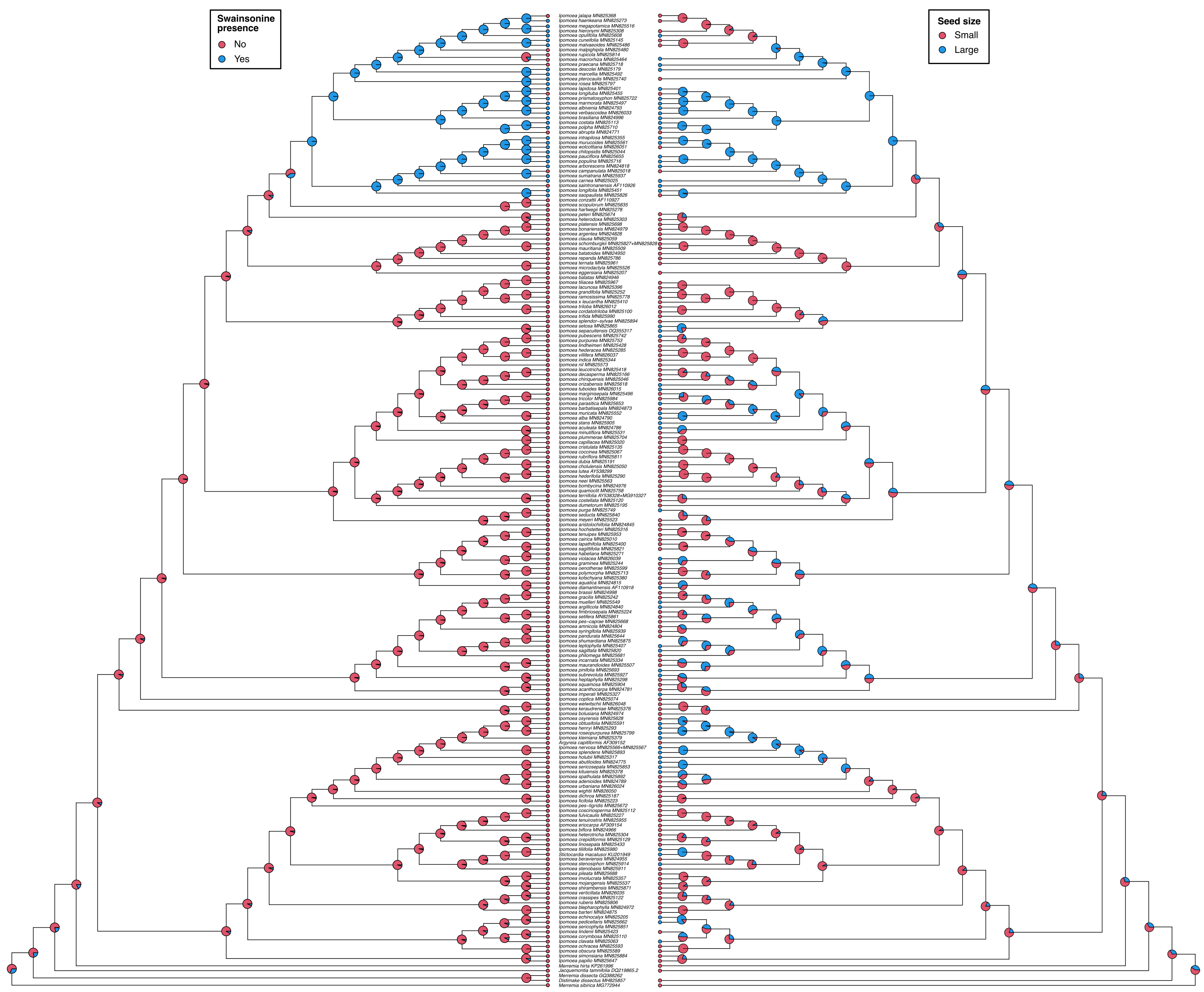
^

**Supplementary Figure 3.** Ancestral state reconstruction phylogenies of swainsonine presence (left) and seed size (right).


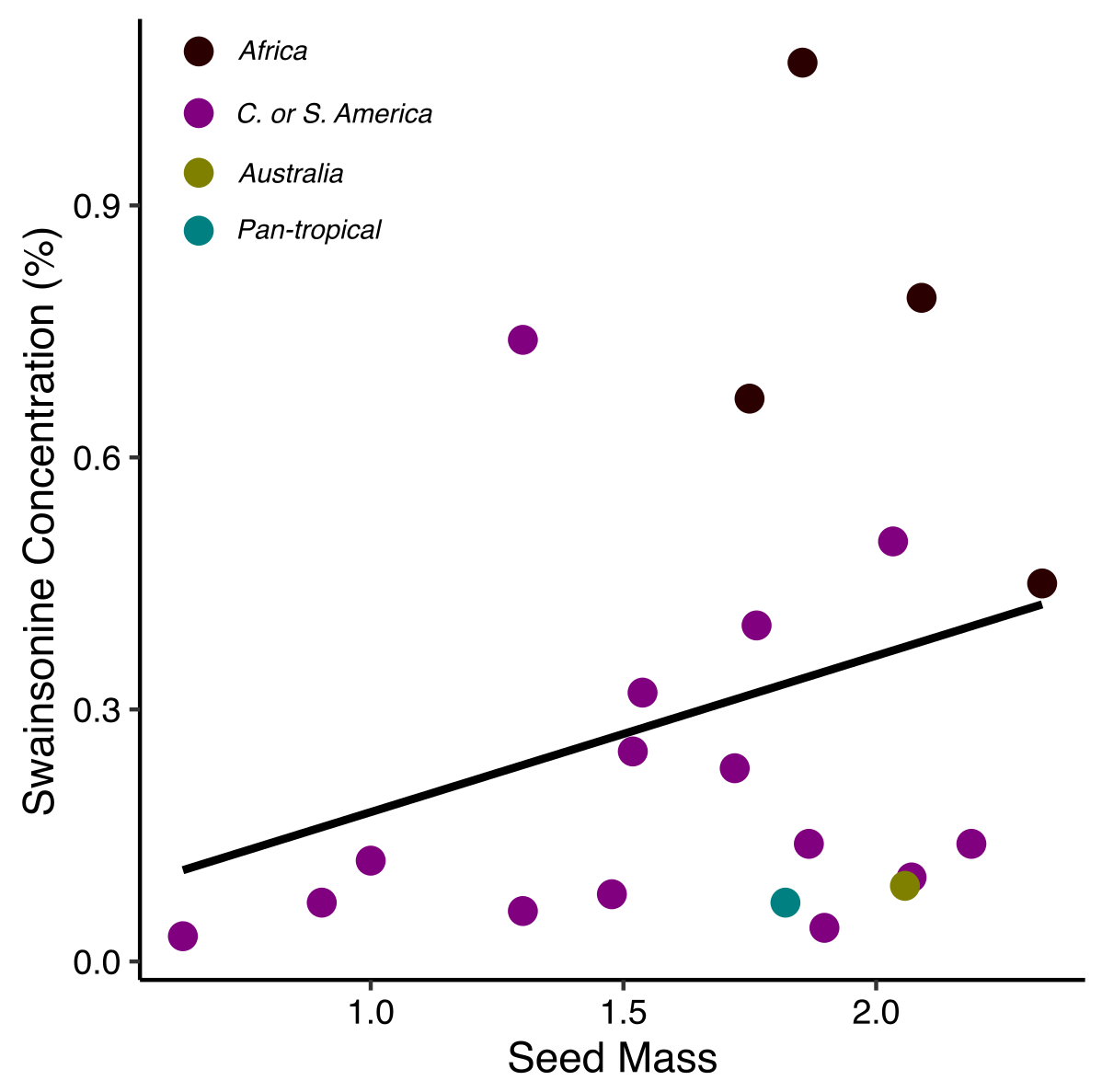


**Supplementary Figure 4**. Linear regression of swainsonine concentration and seed mass in swainsonine-positive species (*p*=0.075, *R^2^*=0.16). C = Central, S = South.

**References**

Schoch, C. L., Sung, G.-H., López-Giráldez, F., Townsend, J. P., Miadlikowska, J., Hofstetter, V., et al. (2009). The Ascomycota tree of life: a phylum-wide phylogeny clarifies the origin and evolution of fundamental reproductive and ecological traits. *Syst. Biol. 58*, 224–239.
